# Application of combined cerebrospinal fluid physicochemical parameters to detect intracranial infection in neurosurgery patients

**DOI:** 10.1186/s12883-020-01781-6

**Published:** 2020-05-27

**Authors:** Tiantian Zhai, Zhong lian Fu, Yan bing Qiu, Qiang Chen, Dong Luo, Kaisen Chen

**Affiliations:** 1 Clinical Laboratory, the First Affiliated Hospital of Nanchang University, Nanchang, 330006 Jiangxi China; 2 School of Public health of Nanchang University, Nanchang, 330006 Jiangxi China; 3 Department of Preschool education and special education, Yuzhang Normal College, Nanchang, 330103 Jiangxi China

**Keywords:** Cerebrospinal fluid, Physicochemical parameters, Discriminant model, Intracranial infection

## Abstract

Routine test of cerebrospinal fluid (CSF), such as glucose concentrations, chloride ion, protein and leukocyte, as well as color, turbidity and clot, were important indicators for intracranial infection. However, there were no models to predict the intracranial infection with these parameters. We collected data of 221 cases with CSF positive-culture and 50 cases with CSF negative culture from January 1, 2016 to December 31, 2018 in the First Affiliated Hospital of Nanchang University, China. SPSS17.0 software was used to establish the model by adopting seven described indicators, and *P* < 0.05 was considered as statistically significant. Meanwhile, 40 cases with positive-culture and 10 cases with negative-culture were selected to verify the sensitivity and specificity of the model. The results showed that each parameter was significant in the model establishment (*P* < 0.05). To extract the above seven parameters, the interpretation model C was established, and C = 0.952–0.183 × glucose value (mmol/L) - 0.024 × chloride ion value (mmol/L)- 0.000122 × protein value (mg/L) - 0.0000859 × number of leukocytes per microliter (× 10^6^/L) + 1.354 × color number code + 0.236 × turbidity number code + 0.691 × clot number code. In addition, the diagnostic sensitivity and specificity of the model were 85.0 and 100%, respectively. The combining application of seven physicochemical parameters of CSF might be of great value in the diagnosis of intracranial infection for adult patients.

## Introduction

Postoperative intracranial infection was an important complication causing adverse prognosis in neurosurgery patients, and the high mortality rate has attracted close attention of clinical doctors [1, 2]. Although strict aseptic operation and usage of prophylactic antibiotics resulted in an apparent decrease of infection rate in recent years [2], the absolute number of deaths caused by postoperative intracranial infection couldn’t be ignored due to the large population. The application of antibiotics was the most important measure for treatment of postoperative intracranial infections, however, when to prophylactically apply certain antibiotics depended on the epidemiology data of hospitals and wards. Because of the long period of bacterial culture and rapid diagnosis of intracranial pathogens in some laboratories, it was of great significance to judge whether there existed intracranial infection with the physicochemical parameters. These common parameters [3–5] included CSF glucose concentration, procalcitonin, lactate, chloride ion, total protein, albumin and leukocyte, as well as color, turbidity, clot formation and Pandy’s test.

However, because of the complexity of intracranial infection, certain errors were existed if we simply depended on one or more parameters to judge intracranial infection [6]. In China, physicochemical parameters in common CSF test included glucose, chloride ion, level of total protein and leukocyte count, as well as color, turbidity, clot and Pandy’s test. In this study, we would screen these parameters to establish Fisher interpretation model for predicting intracranial infection. Meanwhile, some negative and positive samples were selected to verify these comprehensive parameters.

## Materials and methods

### General data

During lumbar puncture, three CSF samples were collected for each patient, including pathogenic culture sample, physical parameter sample and biochemical parameter sample. These CSF physicochemical parameters included glucose levels, chlorine ion, total protein, leukocyte count and color status, turbidity, clot, Pandy’s results. Because total protein had been included, Pandy’s results were excluded for similar value in this study. All data were collected from hospital information system (HIS) from January 1, 2016 to December 31, 2018. Meanwhile, data of 50 patients without intracranial infection during the same period were also collected, which was taken as negative control group. At last, we randomly selected 40 positive cases and 10 negative cases in 2019s to verify the validity of the interpretation model.

### Diagnostic criteria for intracranial infection

The diagnosis of intracranial infection referred to the criteria issued by National Health Commission, and some detailed subjects had also been described by Yu [4]. The criteria were as follows, 1) presence of clinical manifestation of intracranial infection; 2) presence of risk factors, such as HIV/AIDS, hematopoietic stem cell transplant, lymphoid malignancies, neutropenia, hereditary immune defects, patients with drainage or cerebrospinal fluid leakage; 3) leukocyte count > 10 × 10^6^/L; glucose levels < 2.25 mmol/L; chloride ion < 120 mmol/L; total protein > 450 mg/L; 4) positive results for bacteria in cerebrospinal fluid culture. Patients with criteria 4 can be diagnosed individually. Although patients with negative results in CSF culture but positive of the first 3 diagnostic criteria were also highly suspected with intracranial infection, these patients weren’t included in this study. Furthermore, patients with hypoglycemia were excluded in this study even if they had positive intracranial infection. Inclusion criteria for non-bacterial infection group included negative CSF culture results and did not met the first 3 diagnostic criteria. Furthermore, patients did not show headache, fever, neck stiffness and imaging findings did not show any changes about typical intracranial infection.

### Routine pathogenic identification and drug-sensitivity tests

MicroScan WalkAway 96 Plus automatic microbial identification analyzer (Siemens, Germany) was used for bacterial identification and drug sensitive analysis. The instrument was mainly equipped with inoculation water, injection grove and two bacterial plates, PC33 and NC50, respectively. These two bacterial plates were used for the identification and drug-sensitivity tests for Gram-positive cocci and Gram-negative bacilli. The samples in the plates were placed in the MicroScan WalkAway 96 Plus automatic analyzer to incubate for 18–24 h. The instrument could automatically distinguish biochemical reaction and the bacterial growth hole in different gradient of drug concentrations, then the most probable bacterial names and drug susceptible results were obtained correspondingly. Eventually, senior laboratory inspectors reviewed the laboratory operating manual and the rules of the American Association of Clinical and Laboratory Standards to give the bacterial name and drug sensitivity results [7]. Mass spectrometry (Boya Biotechnology Co., Ltd., Beijing, China) was adopted to identify fungi.

### Data acquisition

Experimental data of 221 patients with intracranial infection, CSF samples with traumatic tap and hypoglycemia were excluded, were collected by adopting HIS in this period. The pathogenic strains were obtained by classifying and statistical analysis with excel table. SPSS17.0 software was used for statistics. Three physical indicators (color, turbidity and clot) were adopted with different number codes to represent different levels. In addition, total protein (mg/L), glucose (mmol/L), chloride ion (mmol/L) and leukocyte number (× 10^6^/L) were represented by numerical value. The number code of non-infection group and bacterial infection group was “0” and “1”, respectively. The number code of color was “0” for colorless and transparent liquid, “1” for light yellow and yellow, “2” for red and deep red, “3” for colors other than those described above. The number code of turbidity was “0” for clear and transparent liquid, “1” for slightly turbid and “2” for turbid. The number code of clot was “0” for no clot, “1” for small slot and “2” for big clot. The interpretation standard referred to the operation procedures for clinical examination in China.

The collected data were imported into SPSS17.0 software for statistical analysis. The discriminant model was obtained by Fisher discriminant analysis. Each group of data was statistically analyzed separately, and *P* < 0.05 was considered as statistically significant. At last, these confirmed cases were imported into the interpretation model to verify the validity of the discriminant function.

## Results

### Information of experimental patients and confirmed patients

A total of 221 adults with positive culture were retrospectively collected between January 1, 2016 and December 31, 2018 in the First Affiliated Hospital of Nanchang University, China. The age was ranged from 19 to 71 years old, with a mean age and standard deviation (SD) of 50.61 ± 12.76 years old. Fifty adults with negative culture were also collected during the same period, and the age was ranged from 19 to 69 years, with a mean age and SD of 49.64 ± 11.64 years old. Furthermore, the age of confirmed adults was from 20 to 65 years, with a mean age and SD of 49.34 ± 13.51 years old. The statistical analysis showed that there was no significant difference in age and sex (*P* > 0.05) among the three groups.

### Distribution of pathogens for intracranial infection

A total of 221 pathogens were isolated from postoperative intracranial specimen, including 121 (121/221, 54.8%) gram-positive bacteria, 98 (98/221, 44.3%) gram-negative bacteria and 2 *Cryptococcus neoformans*. Among them, coagulase-negative staphylococci, *Acinetobacter baumannii*, *Klebsiella pneumonia* and *Escherichia coli* accounted for 41.6% (92/221), 12.7% (28/221), 10.0% (22/221) and 4.1% (9/221), respectively. The detailed results were shown in Table 1.

**Table 1:** Distribution and composition of pathogen isolated from postoperative intracranial specimen

Isolated strains	Number (%)
G+ bacteria	121 (54.8)
CNS	92 (41.6)
Streptococcus viride	7 (3.2)
*Staphylococcus aureus*	7 (3.2)
*Enterococcus faecalis*	2 (0.9)
*Enterococcus faecium*	2 (0.9)
Enterococcus raffinosus	1 (0.4)
*Streptococcus pneumoniae*	1 (0.4)
Aerococcus viridans	1 (0.4)
Others	8 (3.6)
G- bacteria	98 (44.3)
Acinetobacter baumannii	28 (12.7)
Klebsiella	22 (10.0)
*Escherichia coli*	9 (4.1)
*Acinetobacter lwoffi*	6 (2.7)
Enteribacter aergenes	4 (1.8)
*Enterobacter cloacae*	4 (1.8)
*Pseudomonas aeruginosa*	2 (0.9)
Haemophilus influenza	1 (0.4)
Citrobacter freundii	1 (0.4)
*Pasteurella multocida*	1 (0.4)
Others	18 (8.1)
Fungi	2 (0.9)
*Cryptococcus neoformans*	2 (0.9)
Total	221 (100.0)

**Note:** CNS: Coagulase-negative staphylococci

### Comparisons between infection group and non-infection group

For the 221 cases with CSF infection, the mean glucose concentration was 2.20 mmol/L, the highest value was 8.36 mmol / L, and the lowest value was 0.01 mmol/L. There were 59 cases with a glucose level below 1.0 mmol / L, 48 cases were showed 1.0 to 2.25 mmol / L, and glucose concentration in patients with fungal infection was between 2.5–3.5 mmol/L. The average CSF glucose concentration in the 50 cases with non-infection was 3.72 mmol/L, the highest value was 8.12 mmol / L, and the lowest value was 2.11 mmol/L. Statistics showed that glucose concentration in non-infection group was significantly higher than that in infection group (*P* < 0.001). It was estimated to be related to the energy consumption in the period of pathogenic growth. The average chloride ion concentration of CSF in the infection group was 118.3 mg / L, the highest value was 167.7 mg/L, and the lowest value was 89.5 mg / L; the average chlorine ion concentration of the non-infection group was 123.2 mg / L, the highest value was 149.2 mg / L, and the lowest value was 104.3 mg / L. Statistics showed that the chloride ion concentration in the non-infection group was significantly higher than that in the infection group (*P* < 0.05), which indicated that the blood-CSF barrier function was reduced after intracranial infection, thereby leading to the loss of CSF chloride ions. The average value of total CSF protein concentration in the infection group was 2587.6 mg / L, the highest value was 10,590 mg / L, and the lowest value was 281 mg / L; while the average value of total protein concentration in the non-infection group was 691.7 mg / L, the highest value was 4118 mg /L, and the lowest value was 72 mg /L. Statistics showed that protein concentration of the infection group was significantly higher than that of non-infection group (*P* < 0.001), indicating that blood-CSF barrier function was decreased and protein inflow was increased after intracranial infection. The average CSF leukocyte concentration in the infection group was 1628.3 × 10^6^/L, the highest value was 42,000 × 10^6^ / L, and the lowest value was 1 × 10^6^ / L. The average leukocyte concentration in the non-infection group was 22.1 × 10^6^ / L, the highest value was 182 × 10^6^ / L, and the lowest value was 1 × 10^6^ / L. Statistics showed that the leukocyte concentration in the non-infection group was significantly higher than that in the infection group (*P* < 0.001). The specimen color of the infection group was mainly yellow or light yellow, while that of the specimen from patient without infection was almost colorless (48/50). The color variance of the two groups were significantly different (*P* < 0.001). Turbidity often appeared in infectious specimen, this study showed that specimens were mainly slightly turbid and turbid in the infection group, while the specimens from patients without infection were mainly clear and transparent (49/50). The appearance of specimens between the two groups was significantly different (*P* < 0.001). In addition, the presence of clots was also related to infection. We found that the specimens of infection patients showed more clots than that of specimens of the non-infection patients. Sixty-three cases were showed clots in the infection group, while only one case was showed clots in the non-infection group. Statistics showed that there was a significant difference between the two groups (P < 0.001).

### Establishment of discriminant model and verification of results

In order to screen the parameters, we separately analyzed seven variables by importing these data into SPSS software 17.0. The results showed that all these parameters were of great value (*P* < 0.05), as shown in Table 2. Fisher discriminant analysis was performed with inclusion of the 7 parameters, and a discriminant model “C” was obtained, C = 0.952–0.183 × glucose value (mmol/L) - 0.024 × chloride ion value (mmol/L) - 0.000122 × protein value (mg/L) - 0.0000859 × number of leukocytes per microliter (× 10^6^/L) + 1.354 × color number code + 0.236 × turbidity number code + 0.691 × clot number code.

**Table 2:** Sensitivity and specificity of Fisher interpretation function

	Calculation results	
	Uninfected cases	Infected cases
Uninfected group (*N* = 10)	10	0
Infected group (*N* = 40)	6	34
sensitivity	85.0% (34/40)	
specificity	100% (34/34)	

Note: 40 positive and 10 negative cases were included. According to the function formula, 10 negative cases were all negative, 34 of 40 positive samples were positive, and 6 were negative. The sensitivity and specificity of this formula was 85.0 and 100%, respectively

In order to verify the validity of the above model, 40 cases with positive culture and 10 cases with negative culture were included in the interpretation model, and the obtained values were compared with the centroid results to determine patients involving in infection group or non-infection group. The result showed that the diagnostic sensitivity and specificity of this model was 85.0 and 100%, respectively.

## Discussion

This study showed that gram-positive bacteria were main infectious bacteria after intracranial surgery, which was consistent with that in the literatures [8, 9]. Our study proved that coagulase-negative staphylococcus (CNS) were the most common bacteria, accounting for 41.6% of all infection pathogens, followed by 3.2% of *Streptococcus viride* and 3.2% of *Staphylococcus aureus*. Most gram-positive bacteria, especially CNS, were common skin colonized [10], which may migrate to surrounding tissues after operation. Intracranial infection caused by environmental bacteria required molecular techniques to identify [11]. The most common gram-negative bacteria were 12.7% of *Acinetobacter baumannii* and 10.0% of *Klebsiella pneumonia* in this study. Because these two bacteria were mainly related to hospital-acquired infections, it was presumed that intracranial infections after neurosurgery were closely related to iatrogenic infections [12, 13]. It was of great significance to prevent cross infection and standardize disinfection of common medical devices [14]. Besides, it was found that some gram-negative bacilli were highly resistant to common antibiotics, such as penicillin, cephalosporins, carbapenems, aminoglycosides and quinolones. Furthermore, more than 80% of staphylococci were methicillin-resistant staphylococcus (MRS) (data no shown). As a result, the rational usage of antibiotics shouldn’t be ignored for subsequent treatment. Because conventional pathogenic culture was time-consuming, we hoped to establish an interpretation model to judge whether patients had CSF pathogenic infections according to seven physicochemical parameters.

The results showed that there were significant differences between pathogenic infection group and non-pathogenic infection group (*P* < 0.05) after comparing these single seven parameters (Table 3). However, the results revealed the specificity and sensitivity of single parameter was low, which couldn’t be well applied in clinical diagnosis. For example, low glucose (less than 2.25 mmol/L) [4] was an important parameter in diagnosing CSF pathogenic infection, the sensitivity and specificity were 60.5% (164/271) and 98.3% (116/118), respectively. CSF white cell content (> 10 × 10^6^/L) [4] was another parameter in diagnosing CSF pathogenic infection, the sensitivity and specificity were 79.0% (214/271) and 92.7% (178/192), respectively.

**Table 3:** Comparison of biochemical indexes and leukocyte number between infecting group and non-infected group

Group	Total number	Glucose (mmol/L)	Chlorine ion (mmol/L)	Protein (mg/L)	Leukocyte (N × 10^6^/L)	Color (N)	Turbidity (N)	Clot (N)
Infectious group	221	AV = 2.214 R: 0.01–8.34	AV = 118.247 R: 89.6–167.5	AV = 2586.922 R:282–10,590	AV = 1627.181 R: 1–42,000	Colorless(54) Yellow (103) Red (51) Other color (13)	Clear and transparent (62) Slightly turbidity (83) Turbid (76)	No clot (158) Slight clot (61) Big clot (2)
Uninfected group	50	AV = 3.710 R:2.12–8.08	AV = 123.456 R:104.6–149	AV = 691.375 R:72–4109	AV = 22.063 R:1–180	Colorless (48) Red (1)	Clear and transparent: (49) Slightly turbidity (1)	No clot (49) Slight clot (1)
*P* value	271	0.000	0.001	0.000	0.000	0.001	0.001	0.02

Note: AV: average; R: range. The *P* value was calculated with hypergeometric distribution of Fisher’s exact test between infected and non-infected group,

*P* value < 0.05 was considered statistically significance

Considering serious lag in the diagnosis of postoperative intracranial pathogenic infection by adopting cultural technique and the shortcomings of single parameter [15], the interpretation model was established depending on the seven parameters. We found that the diagnostic specificity and sensitivity was 85.0 and 100%, respectively, which could well meet the clinical requirements. Of course, reliable diagnosis of intracranial infection also depended on the patient’s symptoms, infection risk factors and imaging evidence. In a word, this model might be helpful for clinical doctors to quickly judge whether there was intracranial infection, so as to timely and effectively use antibiotics.

Of course, there were some limitations of this study. First, the concentration of total protein in CSF divided by total protein in serum can’t reflect the integrity of blood-brain barrier, that was, it can’t reflect pathogen infection if only by globulin elevation. As a result, it was more objective to detect CSF albumin concentration divided by serum albumin concentration, which reflected the blood-CSF barrier function [16]. Secondly, calvarial defects [17] and endoscopic surgery [18] were common in the First Affiliated Hospital of Nanchang University, China. Therefore, when there was bacterial translocation from nasopharynx into the brain, our results showed that the intracranial bacterial culture was positive but these physicochemical parameters were normal, which led to deviation of model establishment. Thirdly, pathogenic culture was positive in the early stage of infection, but there was no difference between the physicochemical results and normal CSF. Fourthly, cases with intracranial infection but negative-culture weren’t included in this study, which led to another biased prediction of interpretation model. Finally, our results came from one hospital, and methods of specimen collection and detecting indicators may be different from those in other hospitals [19], so there were differences in the establishment and application of the model, which needed to be adjusted in combination with the characteristics of the region and the hospital. In conclusion, we had established a model to interpret intracranial infection, although there were various defects, it was valuable for non-early non metastatic infection.

## Conclusion

Levels of glucose, chloride ion, protein and leukocyte, as well as color, turbidity and clot were common physicochemical parameters. The study established a model to predict the intracranial infection. Discriminant model “C” was obtained, C = 0.952–0.183 × glucose value (mmol/L) - 0.024 × chloride ion value (mmol/L)- 0.000122 × protein value (mg/L) - 0.0000859 × number of leukocytes per microliter (× 10^6^/L) + 1.354 × color number code + 0.236 × turbidity number code + 0.691 × clot number code. The diagnostic sensitivity and specificity of this function were 85.0 and 100%, respectively.

## Data Availability

The dataset analyzed during the present study will be available from the corresponding author upon reasonable request based on the guidelines of the Ethics Committee of the First Affiliated Hospital of Nanchang University.
